# Multitrophic and Multidimensional Insights into Biodiversity and Functional Trait Responses to Precipitation Changes in Alpine Grasslands

**DOI:** 10.3390/microorganisms13051011

**Published:** 2025-04-28

**Authors:** Yu Liu, Chengxiang Ding, Zhanjun Quan

**Affiliations:** 1 Institute of Ecology, Chinese Research Academy of Environmental Sciences, Beijing 100012, China; lyu@craes.org.cn; 2 State Key Laboratory of Environmental Criteria and Risk Assessment, Beijing 100012, China; 3 Academy of Animal Husbandry and Veterinary Science, Qinghai University, Xining 810016, China; 2011990016@qhu.edu.cn

**Keywords:** biodiversity, multitrophic, multidimension, functional traits, alpine grassland

## Abstract

Biodiversity and functional traits are increasingly recognized as pivotal factors in regulating ecosystem functioning and stability. However, the patterns and processes of multidimensional biodiversity and functional traits along environmental gradients remain insufficiently understood. In this study, we examined taxonomic, phylogenetic, and functional diversity across multiple trophic levels in 38 alpine grassland sites along a precipitation gradient on the Qinghai–Tibet Plateau. Our findings reveal asynchronous responses of taxonomic, phylogenetic, and functional diversity metrics, as well as functional traits across trophic levels, to variations in precipitation. Soil microbial diversity and functional traits exhibited stronger responses to precipitation changes compared to plant communities, with a tighter coupling observed between microbial diversity and microbial functional traits. Climate and soil properties jointly regulated diversity and functional trait metrics, with the climate exerting greater influence on functional traits and soil properties playing a dominant role in shaping diversity patterns. This study highlights the distinct responses of biodiversity and functional trait attributes to environmental shifts, emphasizing the importance of integrating multidimensional and multitrophic perspectives to advance our understanding of community assembly processes.

## 1. Introduction

Biodiversity is fundamental to the functioning of global ecosystems, as it mediates a range of critical processes (e.g., litter decomposition and nutrient cycling) [1,2]. However, it faces significant threats from climate change, land-use alterations, and habitat loss. As global change continues to unfold, these factors are expected to drive further declines in biodiversity worldwide [3,4]. Understanding the regulating patterns of biodiversity is pivotal for biodiversity conservation [5,6]. Biodiversity studies have traditionally relied on taxonomic species richness due to its simplicity and ease of measurement. However, over the past two decades, the focus has shifted from simply counting species to considering their ecological diversity—specifically, the extent to which species differ in terms of their functions, ecological niches, or evolutionary histories [7]. The most commonly used metrics for evaluating ecological diversity were functional and phylogenetic diversity [8]. The functional diversity focuses on traits that define species’ roles within ecosystems, while the phylogenetic diversity considers their evolutionary relationships. Both diversity metrics offer valuable, complementary insights for conservation efforts [9,10]. Functional diversity metrics provide key information about ecosystem processes, such as resource use and life-history strategies [11], whereas phylogenetic diversity reflects genetic variation, which enhances a community’s ability to adapt to environmental changes [12]. Previous research has demonstrated that the interactions among biodiversity components can exhibit positive, negative, or neutral correlations [8,13]. One well-documented phenomenon is functional redundancy within communities, where the presence of multiple species performing similar ecological roles ensures the maintenance of ecosystem functions despite ongoing species loss [14]. It is important to note that the three dimensions of biodiversity—taxonomic, phylogenetic, and functional—do not always respond synchronously to environmental changes. For instance, De Pauw et al. [9] assessed the taxonomic, phylogenetic, and functional diversity of 225 understory plant communities in European forests and observed that nine different biodiversity metrics exhibited varied responses to altered environmental conditions. Similarly, Belcik et al. [15] compared the responses of biodiversity metrics in bird communities to forest fragmentation in European forests, finding that taxonomic diversity was the most sensitive, while functional diversity was the least responsive. Notably, the shift in focus from species richness to phylogenetic and functional diversity has primarily emerged in studies of aboveground communities [16]. There is limited understanding of biogeographic patterns of taxonomic, phylogenetic and functional diversity of soil microbial communities although they comprise a large fraction of global terrestrial diversity, and exhibit extremely intricate genealogies and diverse ecological functional traits [17,18,19]. 

Biodiversity reflects the functional complementarity among species in terms of ecological niche and life-history strategies, which can maintain the community stability and underpin diverse ecosystem functions [1]. In contrast to diversity theory, the mass ratio hypothesis emphasizes that ecosystem functions are primarily determined by the functional traits of dominant species, such as their strategies for resource use [20]. These functional traits—morphological, physiological, or phenological characteristics—directly influence key aspects of species performance, including growth, reproduction, and survival. For instance, a meta-analysis of global field experiments revealed that arbuscular mycorrhizal fungi play a more critical role in nutrient cycling function compared to species richness [21]. Shifts in the functional composition of communities along environmental gradients can also offer crucial insights into the mechanisms underlying changes in ecosystem functions and services [22,23]. In addition to their role in regulating ecosystem functions, the functional traits of organisms across different trophic levels can be valuable for monitoring biodiversity responses to environmental changes [24]. Since functional trait analysis provides complementary perspectives that extend beyond traditional measures of biodiversity and community composition, integrating biodiversity and functional traits is essential for more accurate predictions of ecosystem dynamics. 

Climate change has been altering precipitation patterns globally [25], with projections for the Qinghai–Tibet Plateau (QTP) indicating an increase in precipitation at a rate of 1.15% per decade [26]. This altered precipitation is expected to cause significant shifts in multiple ecosystem functions by affecting both aboveground and belowground community composition [27,28]. However, the effects of precipitation changes on various dimensions of biodiversity and functional traits in QTP alpine grasslands remain poorly understood. It is recognized that different biodiversity metrics and functional traits may respond differently to climate change [18,29]. Understanding how biodiversity and functional traits across multiple trophic levels respond to precipitation gradients is crucial for predicting the impacts of climate change on the structure and function of both aboveground and belowground communities. Previous research has made valuable contributions to mapping the relationships between functional composition and environmental conditions [30,31], but this work faces three key limitations. First, studies have typically examined the relationship between either taxonomic composition or functional traits and environmental variables, rather than considering both dimensions together [14]. Many studies have focused on a single aspect of biodiversity, such as species richness while neglecting the distinct responses of various biodiversity dimensions and functional traits to climate or soil changes [9]. Third, most studies have focused on a single trophic level, particularly overlooking the functional traits of belowground communities alongside those of aboveground communities [32]. Overcoming these challenges is essential for understanding how changes in precipitation and related soil factors influence different biodiversity attributes and functional traits across alpine grassland types, and for identifying which diversity attributes or functional traits are most sensitive to climate change.

To address these challenges and improve predictions of biodiversity shifts and the drivers of functional biogeography, we conducted a study across 38 sites representing typical alpine grassland types along a precipitation gradient in the Qinghai–Tibet Plateau (QTP). At each site, we collected data on both biodiversity and functional traits for plant and soil microbial communities, as well as measurements of climate and soil physicochemical properties. This study aimed to answer the following key questions: (1) What are the distribution patterns of multiple dimensions of biodiversity and functional traits along the precipitation gradient? (2) How are multiple dimensions of biodiversity and functional traits related to each other? (3) What are the primary drivers of biodiversity and functional traits? We hypothesized as follows: (1) both biodiversity and functional traits would be positively correlated with precipitation, but functional traits would exhibit lower sensitivity to precipitation changes due to functional redundancy; (2) the responses of various biodiversity and functional traits to environmental shifts would be asynchronous, showing positive, negative, or no correlations; and (3) climate factors would have a stronger influence on biodiversity and functional traits than soil properties in the regional scale.

## 2. Materials and Methods

### 2.1. Study Sites and Sampling

**Figure 1:**
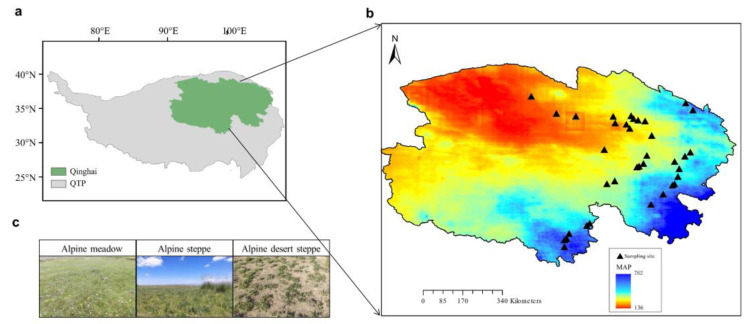
Sampling region (**a**), sampling sites (**b**), and grassland types (**c**) across the Qinghai–Tibet Plateau (QTP). In panel (**b**), colors indicate different levels of annual mean precipitation.

A field survey was conducted at 38 sites along a transect in the alpine grasslands of the QTP, covering a temperature range of −2.29 to 7.23 °C and annual precipitation ranging from 136 to 762 mm (Figure 1, Table 1). Fieldwork took place from July to August 2020, covering three grassland types: alpine meadow, alpine steppe, and alpine desert steppe. At 29 sites with only an herbaceous layer, three 0.5 m × 0.5 m quadrats were placed in a 25 m^2^ area. At 9 sites with both shrub and herbaceous layers, three 2 m × 2 m shrub quadrats and three 0.5 m × 0.5 m herbaceous quadrats were established within the shrub quadrats, also within a 25 m^2^ area. For each quadrat, data on vegetation cover, species number, coverage, and abundance were recorded. Leaf samples for functional trait analysis were collected from dominant species, with 3–5 individuals per species and three replicates per site. Soil samples were collected from three quadrats at each site by extracting five 0–10 cm soil cores per quadrat, which were pooled into composite samples for subsequent microbial and soil physicochemical analysis. Microbial samples were stored at −20 °C for DNA extraction, and soil samples were kept at 4 °C for physicochemical analysis.

### 2.2. The Plant Traits, Soil Physicochemical Properties and Climate Data Collection

Dominant species within each quadrat were identified based on their relative cover and selected for functional trait measurements. Detailed information on vegetation types and the dominant species sampled at each site is provided in Table 1. Plant functional traits were measured following the standardized protocol of Cornelissen et al. [33]. For plant height, the maximum height of 10 randomly selected individuals per species was measured in the field using a ruler or measuring tape, and the mean value was calculated. For each species, 5–10 mature leaves were collected, fully hydrated in water for 2 h, and gently blotted with filter paper to remove surface moisture. Leaf area was determined using ImageJ software (v1.8.0; National Institutes of Health, Bethesda, MD, USA), after which the leaves were oven-dried at 65 °C for 48 h to obtain dry weight. Specific leaf area (SLA) was calculated as the ratio of leaf area to dry weight. Leaf carbon (LC) and nitrogen (LN) contents were quantified using an elemental analyzer (Vario EL cube, Elementar Analysensysteme GmbH, Langenselbold, Germany). Soil water content (SWC) was measured by drying soil samples at 105 °C for 24 h, while soil bulk density (SBD) was determined using a 100 cm^3^ ring cutter to extract undisturbed soil, which was subsequently dried at 105 °C for 48 h. Soil pH was determined by mixing fresh soil with water in a ratio of 1:5, and measurements were taken using a Sartorius pH meter (PB–10, Sartorius Corporate Administration GmbH, Göttingen, Germany). Soil texture was analyzed using the sedimentation method with a hydrometer [34,35]. Soil organic carbon (SOC) was measured with the K_2_Cr_2_O_7_ oxidation-reduction titration, total nitrogen (TN) was determined with the Kjeldahl digestion methods. Total phosphorus (TP) and total potassium (TK) were measured using the flame photometry method following digestion with sodium hydroxide [36]. Dissolved organic carbon (DOC) was determined using TOC analyzer (TOC-L CPN, Shimadzu, Kyoto, Japan). Microbial biomass carbon (MBC) and microbial biomass nitrogen (MBN) were measured using chloroform fumigation–extraction method [37]. Five climate variables were extracted: mean annual temperature (MAT), mean annual precipitation (MAP), potential evapotranspiration (PET), standardized precipitation-evapotranspiration index (SPEI) and solar radiation (SR). MAT data were sourced from the Dataset of Surface Climate Normals from 219 Chinese International Exchange Stations (1991–2020), while MAP data were derived from the Climate Hazards Center InfraRed Precipitation with Station data. PET and SR data were obtained from the ERA5-Land Monthly Aggregated dataset [38], and SPEI data from The Global SPEI database. Climate data for each site were acquired using geographic coordinates, and to minimize errors due to varying sampling times, a 20-year mean value (2001–2020) was used where possible, in line with common practice in global analyses [39].

### 2.3. DNA Extraction, Sequencing, and Bioinformatics Analysis

Soil DNA was extracted from 0.25 g frozen soil samples using the PowerSoil DNA Isolation Kit (MO BIO Laboratories, Carlsbad, CA, USA) following the provided instruction manual. Quantification of extracted DNA was performed using a NanoDrop 2000 Spectrophotometer. The amplification of bacteria 16S ribosomal RNA genes (including bacteria and archaea) and fungal internal transcribed spacer regions was performed using specific primer pairs: 515F/806R for bacteria and ITS1F/ITS2 for fungi, as outlined by [40]. The polymerase chain reaction was conducted using a Mastercycler Gradient (Eppendorf, Hamburg, Germany) following the manufacturer protocols. The resulting paired-end reads, approximately 250 base pairs in length, were generated using the Illumina MiSeq platform. Using the UNOIS3 algorithm of USEARCH to filter out low-quality reads and chimeras, generating zero-radius operational taxonomic units (ZOTU) [41]. Taxonomic classification of ZOTUs was achieved using the QIIME2 naive Bayes classifier trained on the SILVA rRNA database (v 132) and the UNITE database for bacteria and fungi, respectively. To enhance the robustness of the analysis, only ZOTUs present in at least 20% of the total samples and containing a minimum of 4 counts in each sample were retained. All subsequent data analysis will be conducted based on the filtered dataset. FastTree2 based on the approximate maximum likelihood method was used to construct phylogenetic trees for bacteria and fungi, respectively [42].

### 2.4. Functional Traits of Plant, Bacteria and Fungi

Four measured plant functional traits were used to calculate the plant functional diversity and community weighted mean (CWM) based on the Gower distance of functional traits using the FD package [43]. Soil fungal functions were obtained from the predicted results of FungalTraits [44]. Soil bacterial functions were estimated based on the predicted results of FAPROTAX and filter the functions related to nutrient cycling [45]. To ensure ecological relevance and reliability, we selected high-confidence functions with relatively high predicted abundance across samples. The retained bacterial functional traits included: cellulolysis, hydrocarbon degradation, methanotrophy, denitrification, nitrogen fixation, and nitrate reduction. Fungal functional traits included: arbuscular mycorrhizal, foliar endophyte, mycoparasite, dung saprotroph, ectomycorrhizal, plant pathogen, dung saprotroph and soil saprotroph.

### 2.5. Multiple Attributes of Diversity 

We calculated taxonomic, phylogenetic and functional diversity metrics to represent multiple attributes of biodiversity in each site. The taxonomic α diversity of plants, bacteria, and fungi was represented by richness index of plant species and ZOTUs of bacteria and fungi. For fungi and bacteria, we constructed phylogenetic tree using aligned and preprocessed sequences. For estimating plant phylogenetic diversity, we initially standardized the scientific names of these plant species using the U.Taxonstand package [46]. Following this, we constructed plant phylogenetic trees using the V.PhyloMaker2 package [47]. Faith’s phylogenetic diversity was subsequently computed based on the generated phylogenetic trees. The richness of fungal functional traits was used as a proxy of fungal function diversity. The bacteria functions were obtained from the predicted results of PICRUST2 [48]. The richness index of enzyme metabolic pathways on the basis of Kyoto Encyclopedia of Genes and Genomes orthologs (KOs) represented the bacteria’s functional diversity. Functional dispersion (FDis) quantifies the breadth of functional roles across species and shows higher stability to outliers, thus we used FDis as the proxy of plant functional diversity. 

### 2.6. Data Analysis

Ordinary least square models were used to estimate the linear relationships between environmental factors and biological components. Mantel’s correlation analysis was used to evaluate the correlations between the Euclidean distance of environmental factors and Bray–Curtis dissimilarity of biodiversity and functional traits. Pearson correlation analysis was used to evaluate the correlations between environmental factors and correlations between biodiversity and functional traits. Variance partitioning analysis (VPA) was employed to assess the relative contributions of climate and soil properties on biological components. To mitigate the strong collinearity among variables, we removed drivers with Spearman’s ρ2 > 0.70 utilizing the ‘varclus’ function from the Hmisc package. To identify the key predictors in driving biological components, we conducted a random forest analysis for each indicator of biological components using the ‘randomForest’ function of the rfPermute package [49]. This approach was chosen due to the non-normal distribution of the data. All statistical analyses were performed using R software (v.4.0.2).

## 3. Results

### 3.1. Responses of Multiple Dimensions Biodiversity of Plant, Soil Bacteria and Soil Fungi to Precipitation Shifts

**Figure 2:**
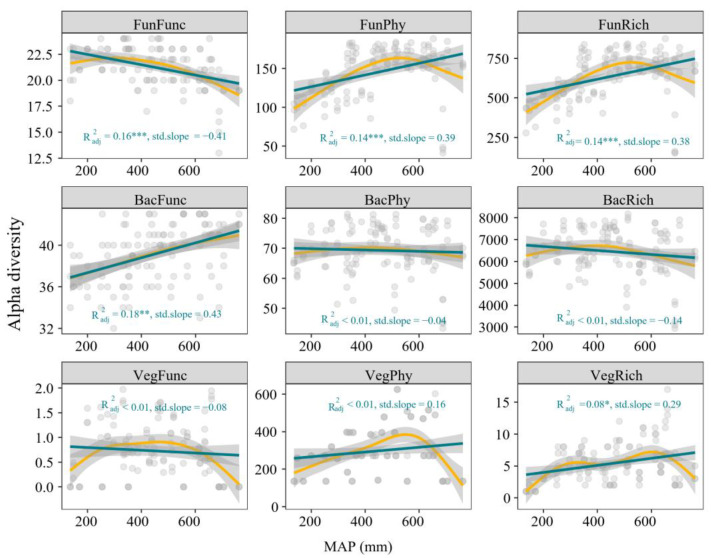
Multiple dimensions of plant and soil microbial diversity along the precipitation gradient. FunFunc, FunPhy, and FunRich represent the functional, phylogenetic, and taxonomic diversity of fungi, respectively. BacFunc, BacPhy, and BacRich represent the functional, phylogenetic, and taxonomic diversity of bacteria, respectively. VegFunc, VegPhy, and VegRich represent the functional, phylogenetic, and taxonomic diversity of plants, respectively. Green lines indicate ordinary least squares (OLS) regression, and yellow lines indicate generalized additive model (GAM) regression. * *p* < 0.05, ** *p* < 0.01, *** *p* < 0.001.

The multiple dimensions of diversity exhibited distinct relationships with precipitation. The phylogenetic diversity and richness of fungi, bacteria functional diversity and plant richness responded significantly and positively in a linear manner to increasing precipitation, fungal functional diversity showed negative significant relationships with increasing precipitation, bacterial phylogenetic diversity and richness, as well as plant phylogenetic and functional diversity, showed no significant response to increasing precipitation (Figure 2). 

### 3.2. Responses of Multiple Dimension Functional Traits of Plant, Soil Bacteria and Soil Fungi to Precipitation Shifts

**Figure 3:**
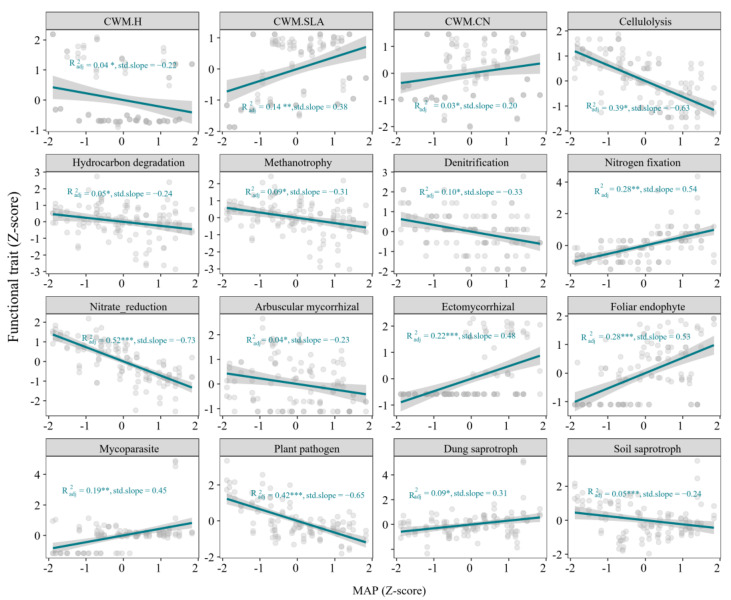
Functional traits of plant, soil bacteria and soil fungi along the precipitation gradient. CWM.H, CWM.SLA, CWM.CN are the community-weighted mean of plant height, plant specific leaf area and C:N ratio of plant leaf, respectively. * *p* < 0.05, ** *p* < 0.01, *** *p* < 0.001.

Precipitation showed significant linear relationships with all plant, bacterial, and fungal trait metrics (*p* < 0.05, Figure 3). Specifically, CWM.SLA, CWM.CN of plant functional traits, nitrogen fixation of bacteria function traits, ectomycorrhizal, foliar endophyte, mycoparasite, dung saprotroph, and soil saprotroph among fungal functional traits showed significant positive relationships with increased precipitation, while cellulolysis, hydrocarbon degradation, methanotrophy, denitrification, nitrate reduction, arbuscular mycorrhizal, plant pathogen showed negative relationships with increased precipitation.

### 3.3. Responses to Distance Metrics of Biodiversity and Functional Traits with Differences in Precipitation

**Figure 4:**
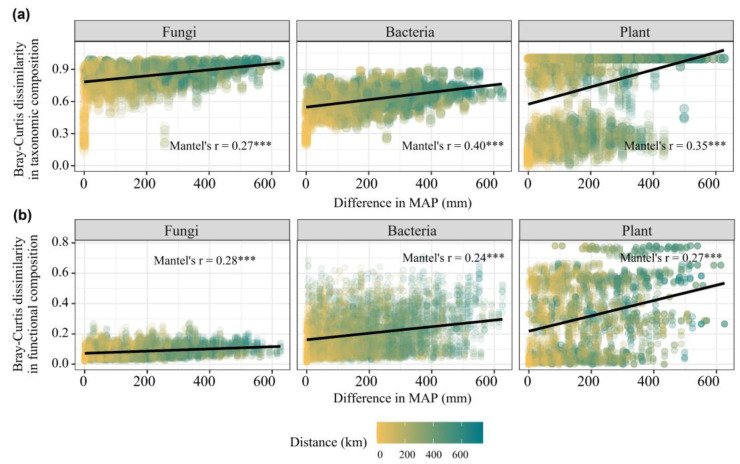
The Mantel correlations between distance matric of taxonomic and functional compositions of plant (**a**), soil bacterial and fungal communities (**b**) and differences in MAP. *** *p* < 0.001.

The Bray–Curtis dissimilarity of taxonomic and functional compositions of plant, bacterial and fungal communities showed significant Mantel correlations (Mantel’s r = 0.24–0.4, *p* < 0.05) with the differences in precipitation (Figure 4a,b), indicating clear linear decay in community similarity with an increasing difference in precipitation.

### 3.4. Relationships Between Biodiversity and Functional Traits 

**Figure 5:**
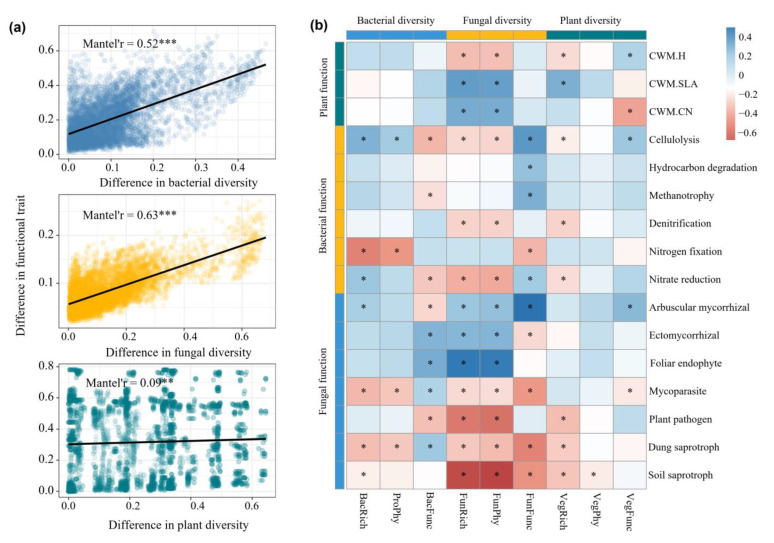
The link between biodiversity and functional traits: (**a**) Mantel correlations between distance matric of functional traits and biodiversity of plant, soil bacterial and fungal communities; (**b**) the Pearson correlations between biodiversity and functional traits. FunFunc, FunPhy and FunRich are the functional, phylogenetic and taxonomic diversity of fungi, respectively. BacFunc, BacPhy and BacRich are the functional, phylogenetic and taxonomic diversity of bacteria, respectively. VegFunc, VegPhy and VegRich are the functional, phylogenetic and taxonomic diversity of plants, respectively. CWM.H, CWM.SLA, CWM.CN are the community-weighted mean of plant height, plant specific leaf area and C:N ratio of plant leaf, respectively. * *p* < 0.05, ** *p* < 0.01, *** *p* < 0.001.

We found significant Mantel correlations between the Bray–Curtis dissimilarity of plant, soil fungal, and soil bacterial diversity and the Bray–Curtis dissimilarity of their functional traits. Soil bacterial and fungal communities exhibited substantially higher Mantel correlations (0.52 and 0.53, respectively) compared to plants, indicating stronger associations between microbial diversity and functionality (Figure 5a). Pearson correlation analysis revealed both positive, negative, and non-significant relationships between biodiversity and functional traits of all taxa. Fungal diversity indicators, including FunFunc, FunPhy, and FunRich, exhibited more significant correlations with the functional traits (Figure 5b). 

### 3.5. Environmental Drivers of Biodiversity and Functional Traits 

**Figure 6:**
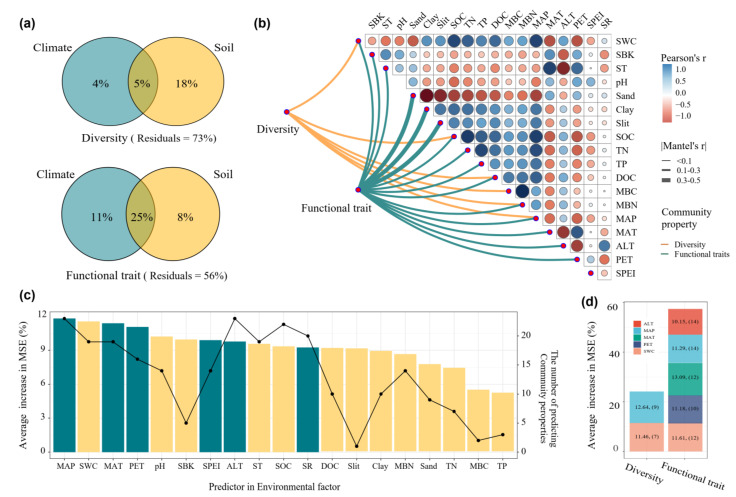
The primary drivers of biodiversity and functional traits: (**a**) the VPA of functional traits and biodiversity; (**b**) the Mantel correlations analysis between functional traits and biodiversity and environmental factors; (**c**) the predictors of biodiversity and functional traits based on random forest model; (**d**) the primary drivers of biodiversity and functional traits based on random forest model. Environmental variables include: soil water content (SWC), soil bulk density (SBD), soil pH, soil organic carbon (SOC), total nitrogen (TN), total phosphorus (TP), total potassium (TK), dissolved organic carbon (DOC), microbial biomass carbon (MBC), microbial biomass nitrogen (MBN), clay content, silt content, sand content, mean annual temperature (MAT), mean annual precipitation (MAP), potential evapotranspiration (PET), altitude (ALT), and the standardized precipitation evapotranspiration index (SPEI).

VPA analysis revealed that the combined effects of climate and soil properties explained 5% of the variation in biodiversity and 25% in functional traits, respectively. Soil properties had a stronger direct effect than climate factors in explaining biodiversity variation (18% vs. 4%), but a weaker direct effect than climate factors in explaining functional trait variation (Figure 6a). Mantel tests showed that biodiversity was significantly correlated with most soil factors but only with MAP among climate variables. Functional traits were significantly correlated with both most soil and climate factors (Figure 6b). Random forest analysis indicated that MAP had the highest predictive accuracy for both biodiversity and functional traits, resulting in a 13.5% increase in mean squared error (MSE). SWC was the second-best predictor overall and the most significant soil property predictor, with an MSE increase of 12.9%. MAP was the most versatile predictor, influencing all 25 biological components (Figure 6c). After filtering environmental factors that explained more than 60% of the variation in biodiversity and functional trait indicators, we found that biodiversity was mainly influenced by MAP and SWC, while functional traits were primarily regulated by multiple environmental factors, including ALT, MAP, MAT, PET and SWC.

## 4. Discussion

### 4.1. The Distinct Response Patterns of Multidimensional Biodiversity and Functional Traits to Precipitation Change

In general, we found that increasing precipitation resulted in distinct responses of diversity metrics and functional trait metrics of aboveground and belowground communities (Figure 2 and Figure 3). The linear regression models showed that plant and fungal functional diversity decreased as precipitation increased, while their species and phylogenetic diversity increased (Figure 2). This suggests that wetter conditions might support a broader range of plant and fungal lineages but not necessarily a greater variety of functions, possibly due to functional redundancy or the dominance of certain adaptive traits in moist environments [50]. In contrast, bacterial functional diversity increased with precipitation, but their species and phylogenetic diversity remained stable (Figure 2). This may indicate that bacterial communities adapt functionally to wetter conditions through increased metabolic or ecological functions (e.g., the abundance or expression of functional genes), rather than via changes in species composition or phylogenetic structure [51]. Moreover, large-scale investigation in Tibetan grassland demonstrated bacterial diversity contributed larger to the variation of soil multifunctionality than fungi and plant diversity [52]. These results suggest that bacterial communities may play a stabilizing role in ecosystem functioning as environmental conditions shift. We also observed asymmetric response patterns of various functional traits to precipitation, highlighting adaptive functional shifts across taxa in response to moisture availability and reflecting their diverse ecological strategies. For example, the positive correlations of CWM.SLA and CWM.CN with MAP suggest that wetter conditions favor resource-acquisitive traits, where plants optimize growth by producing leaves with higher SLA and nutrient efficiency, which is common in environments with sufficient water [53]. In contrast, the negative association of CWM.H with MAP might indicate reduced investment in growth for light capture in wet environments, where water availability outweighs competition for light [54]. 

Although distinct responses of multiple diversity attributes and functional traits to precipitation, we found consistent positive Mantel correlations between diversity and functional trait distance metrics with precipitation distance (Figure 3), indicating that spatial patterns of both diversity and functional traits are significantly aligned with variations in precipitation across sites. This alignment implies that even though different taxa or functional groups respond uniquely to changes in precipitation, overall community structures—both in terms of who is present (diversity) and how they function (traits)—are shaped in parallel by precipitation gradient [55]. The results of the random forest model also demonstrated that MAP was the primary driver of both biodiversity and functional traits (Figure 6c,d). Precipitation manipulation experiment conducted in forest [56], desert [57], and steppe ecosystems [58] have also demonstrated that changes in water-use strategies, induced by precipitation variation, along with alterations in plant physiological activities, regulated both biodiversity and functional traits. These findings underscore precipitation as a key determinant of ecosystem structure and function in the QTP alpine grasslands, highlighting how variations in water availability can synchronize ecological responses across different biological dimensions.

### 4.2. The Significant Correlations Between Biodiversity and Functional Traits

The use of multiple facets of diversity (taxonomic, phylogenetic and functional diversity) of multiple trophic levels (plant, soil bacteria and fungi) provided different, complementary information on community assembly mechanisms in alpine grassland across QTP. From the Pearson correlation matrix, it was clear that diversity metrics among different trophic levels had weak associations while diversity metrics within the same trophic level had stronger correlations (Figure S1), indicating decoupled relationships between different trophic and low redundancy across them [32]. In addition, high phylogenetic conservation was observed in soil microbial communities rather than in plant communities. Taxonomic diversity of both bacteria and fungi was significantly correlated with their phylogenetic richness (r = 0.97 and 0.98, *p* < 0.001), while plant phylogenetic diversity had weaker correlations with taxonomic diversity but stronger correlation with functional diversity (r = 0.49, *p* < 0.001, Figure S1). These results indicate that microbial communities may exhibit niche differentiation, where phylogenetic diversity reflects ecological adaptability [59]. The lack of correlation with functional diversity implies that microbial species often overlap functionally, meaning phylogenetic diversity might be more critical for ecosystem stability in microbial communities [60]. For plants, the independence of species composition and phylogenetic structure suggests that patterns of plant diversity are distinct from those of belowground microbiota diversity [61]. Phylogenetically diverse plants exhibit different ecological roles and low functional redundancy [62]. However, Lososová et al. [63], who sampled 32 large cities across ten countries, found significant but weak positive relationships between phylogenetic diversity and overall functional diversity. Similarly, Mazel et al. [64], using data on ecologically relevant traits from over 15,000 vertebrate species, concluded that maximizing phylogenetic protection alone is insufficient to preserve functional diversity. Thus, biodiversity management should address multiple dimensions of diversity separately to capture the shifts in biodiversity and enhance ecosystem function and resilience effectively. 

We also observed significant correlations between biodiversity metrics of plants, soil fungi, and soil bacteria, and different attributes of ecological functional traits (Figure 5b). Among them, fungal diversity, including (FunFunc, FunPhy, FunRich) exhibited the strongest correlations with functional traits compared to plants and soil bacteria (Figure 5b). This result aligns with the known role of fungi in mediating key ecosystem functions such as nutrient cycling, decomposition, and symbiotic relationships with plants [65,66]. Fungi are integral to soil ecosystems, often acting as keystone species that influence various ecological processes. Their diverse functional traits—ranging from nutrient acquisition strategies to stress tolerance—could explain why fungal diversity has the most substantial link with functional traits [67]. For example, Qiu et al. [21] conducted a meta-analysis of 322 data points from 36 studies to assess the effect of arbuscular mycorrhizal fungi (AMF) inoculum on N and P cycles. Their results showed that AMF inoculation reduced soil NO_3_^−^-N and TP losses by 32% and 21%, respectively. The mitigation effects of AMF on N and P loss increased with higher rates of AMF root colonization. The research in boreal forest soil [68] and coastal salt marsh [69] found that fungal richness contributes more to multifunctionality than bacteria. Moreover, a global field survey reported that fungal phylotype richness plays a key role in driving the stability of plant productivity in terrestrial ecosystems [70]. These results underscore the critical role of fungal diversity in maintaining ecosystem stability and functioning, especially in soils where fungi drive critical biogeochemical cycles. Soil bacteria diversity, especially bacteria functional diversity exhibited a notable correlation with functional traits (Figure 5b). A study by Jia et al. [71] found that fungal diversity enhanced multifunctionality in soils with low pathogen abundance, while bacterial diversity drove multifunctionality regardless of pathogen levels. Similar findings were reported in degraded grasslands, where the soil bacterial community contributed to the temporal stability of plant productivity through increased network complexity [72]. Soil bacteria’s functional traits, including metabolic pathways and stress resistance mechanisms, directly correlate with their ability to sustain ecosystem processes, which may explain their strong associations with functional traits [73]. In contrast to soil microbial communities, plant taxonomic diversity rather than functional diversity exhibited stronger correlations with ecological functional traits (Figure 5b). The stronger correlation between plant taxonomic diversity and functional traits could be due to the relatively wide range of functional roles that plants can fulfill within an ecosystem [74]. For example, different plant species may exhibit highly divergent functional traits related to photosynthesis, nutrient uptake, and water use efficiency [75]. In contrast, the more specialized functional traits of microbes (such as those related to nutrient cycling and decomposition) can lead to a tighter coupling between microbial diversity and ecosystem processes [60]. This underscores the importance of plant diversity in shaping ecosystem processes through species-specific traits, particularly in more stable or minimally disturbed ecosystems. 

### 4.3. The Different Environmental Drivers of Biodiversity and Functional Traits

Our findings indicate that environmental variables, particularly soil and climate, play a dominant role in shaping the biological attributes of organisms, with specific differences between the drivers of functional traits and taxonomic diversity. The VPA analysis showed that climate and soil properties explained 27% and 44% variations of diversity and functional traits, respectively (Figure 6a). The Mantel correlation analysis revealed that functional traits had much more significant Mantel correlations with climate and soil factors compared to diversity (Figure 6b). These results suggest that the distribution patterns of ecological functional traits of taxa are more tightly linked to environmental variation than biodiversity [76]. The results of the VPA and random forest model showed that climate was a more significant determinant of functional traits than biodiversity (Figure 6c). Climate factors, such as temperature and precipitation, regulate plant growth patterns, resource allocation, and phenological traits (e.g., leaf morphology, seed dispersal timing, or drought tolerance), as well as microbial metabolic activity. For instance, in regions with extreme temperature fluctuations or arid conditions, plants may develop functional traits such as drought resistance or enhanced water-use efficiency, which are direct adaptations to climate pressures [77]. Similarly, soil microorganisms often exhibit functional trait plasticity in response to changing climatic conditions, with different microbial communities evolving distinct functional traits suited to specific climate regimes [78,79]. Therefore, the clear link between functional traits and climate in this study highlights the role of climate as a key ecological filter, determining the capacity of organisms to adapt to and persist under global climate change [80]. 

In contrast to the environmental drivers of functional traits, our findings suggest that biodiversity was more strongly influenced by soil factors (Figure 6). Soil properties such as soil water content, nutrient availability and organic carbon content directly influenced the composition and abundance of species in both plant and microbial communities. Research in alpine grasslands has reported that higher soil organic carbon and total nitrogen contents were associated with greater plant diversity in alpine meadows and alpine steppe compared to alpine desert ecosystems [81]. Zhang et al. [82], using broad-scale national forest inventory data from Canada, found that climatic factors had a weaker influence on plant richness in the understorey strata compared to the canopy layer. Total species richness was most strongly correlated with soil conditions. For the soil microbiome, a global-scale meta-analysis reported that climate factors do not consistently lead to a reduction in microbial diversity. Instead, climate-induced shifts in microbial diversity are primarily driven by changes in soil pH [83]. The mechanisms by which climate and soil factors influence biodiversity tend to be complex, and relative importance of them are typically context-dependent and scale-dependent in most studies. Furthermore, incorporating edaphic variation into climate change research can enhance the predictive accuracy of species distribution models, help identify potential climate refugia, and pinpoint species with adaptive traits that may buffer them from the impacts of climate change [84].

## 5. Conclusions

Integrating multiple dimensions and multitrophic biodiversity is essential for assessing community assembly processes. Using a broad precipitation gradient and representative alpine grassland transects, we demonstrate that various diversity attributes and functional traits exhibit distinct responses to shifts in precipitation and are influenced by different environmental drivers. Biodiversity and functional traits are not interchangeable but provide complementary and critical insights into ecosystem dynamics. Future studies should address this complexity by incorporating intraspecific trait variation to enhance our understanding of functional biodiversity patterns and the underlying mechanisms of ecosystem stability. As grassland ecosystems on the Qinghai–Tibet Plateau are likely to face increased precipitation fluctuations in the future, we anticipate a more pronounced decoupling of different dimensions of biodiversity. 

## Figures and Tables

**Table 1:** Details of sampling points.

Site	MAP (mm)	MAT (°C)	Altitude	Vegetation Type	Dominant Species
XW	519.3	−1.25	4196	Cyperaceae	Kobresia tibetica, Ligularia virgaurea, Tibetia himalaica
JLL	429.18	−1.11	4204	Cyperaceae	Kobresia tibetica, Leontopodium leontopodioides, Pedicularis kansuensis
ZK	569.83	−0.98	3933	Cyperaceae	Kobresia tibetica, Leontopodium leontopodioides, Pedicularis kansuensis
JG	635.69	0.53	3406	Cyperaceae	Kobresia tibetica, Potentilla multiceps, Tibetia himalaica
NQ3	555.96	−1.55	4153	Cyperaceae	Kobresia tibetica, Potentilla multiceps, Leontopodium leontopodioides
EB	704.86	−2.29	3598	Rosaceae	Potentilla multiceps, Ligularia virgaurea, Tibetia himalaica
NQ2	614.54	2.24	4329	Cyperaceae	Kobresia tibetica, Potentilla multiceps, Tibetia himalaica
XW1	509.11	0.72	3803	Cyperaceae	Kobresia tibetica, Ligularia virgaurea, Potentilla multiceps
DW1	687.74	−0.76	3933	Cyperaceae	Kobresia tibetica, Potentilla multiceps, Tibetia himalaica
DWS	687.74	−0.76	3933	Cyperaceae	Kobresia tibetica, Potentilla multiceps, Tibetia himalaica
NQ4	555.96	−1.55	4144	Cyperaceae	Kobresia tibetica, Potentilla multiceps, Tibetia himalaica
NQ1	659.6	5.13	3632	Cyperaceae	Kobresia tibetica, Potentilla multiceps, Pedicularis kansuensis
ELSK	481.82	−2.67	4462	Rosaceae	Potentilla multiceps, Kobresia tibetica, Tibetia himalaica
JXLC	617.49	0.37	3927	Cyperaceae	Kobresia tibetica, Potentilla multiceps, Tibetia himalaica
DR	652.4	−0.33	4128	Cyperaceae	Kobresia tibetica, Potentilla multiceps, Tibetia himalaica
MD1	366.5	0.82	4224	Cyperaceae	Kobresia tibetica, Potentilla multiceps, Tibetia himalaica
MD2	386.62	−0.04	4229	Cyperaceae	Kobresia tibetica, Potentilla multiceps, Tibetia himalaica
WQ	420.02	−0.53	4243	Cyperaceae	Kobresia tibetica, Potentilla multiceps, Tibetia himalaica
GD	461.81	7.23	2508	Poaceae	Achnatherum Splendens, Peganum multisectum, Artemisia frigida
DW	634.58	3.34	3252	Poaceae	Achnatherum Splendens, Artemisia frigida, Poaceae luteolus
GN	513.61	4.07	3345	Cyperaceae	Kobresia tibetica, Potentilla multiceps, Tibetia himalaica
GN2	447.28	5.56	3223	Poaceae	Achnatherum Splendens, Artemisia frigida, Poaceae luteolus
MY	762.16	1.51	3090	Rosaceae	Potentilla fruticosa
GN4	470.14	4.07	3282	Cyperaceae	Kobresia tibetica, Potentilla multiceps, Thermopsis lanceolata
XH	438.24	1.54	3737	Cyperaceae	Kobresia tibetica, Potentilla multiceps, Poaceae luteolus
TJ2	334.44	3.54	3231	Poaceae	Achnatherum Splendens, Leymus Chinensis
TJ3	342.05	3.17	3304	Cyperaceae	Kobresia tibetica, Ligularia virgaurea, Tibetia himalaica
TJ4	354.73	3.51	3345	Cyperaceae	Kobresia tibetica, Ligularia virgaurea, Tibetia himalaica
TJ5	351.47	2.23	3432	Cyperaceae	Kobresia tibetica, Ligularia virgaurea, Tibetia himalaica
CK1	331.66	2.93	3300	Poaceae	Achnatherum Splendens, Polygonum viviparum
CK2	254.54	4.33	3124	Poaceae	Elymus nutans, Leymus Chinensis
CK3	274.77	4.93	3300	Poaceae	Elymus nutans, Leymus Chinensis
GLM	136	4.12	3882	Chenopodiaceae	Peganum multisectum
DL	296.73	4.41	3195	Chenopodiaceae	Polygonum viviparum, Artemisia frigida
DLH	170.59	4.5	2806	Chenopodiaceae	Peganum multisectum
DLH2	147.9	4.64	3622	Poaceae	Peganum multisectum, Artemisia frigida
WL1	199.96	4.09	3451	Poaceae	Achnatherum Splendens, Artemisia frigida,
WL2	249.59	2.38	3466	Poaceae	Achnatherum Splendens, Artemisia frigida

## Data Availability

The data presented in this study are available upon request from the corresponding author. The data are not publicly available due to privacy restrictions.

## Supplementary Materials

The following supporting information can be downloaded at: https://www.mdpi.com/article/10.3390/microorganisms13051011/s1, Figure S1: The correlations between multiple dimensions diversity.
