# Supplementary material for: Multitrophic and Multidimensional Insights into Biodiversity and Functional Trait Responses to Precipitation Changes in Alpine Grasslands
Source: Microorganisms. 2025 Apr 28;13(5):1011. doi: 10.3390/microorganisms13051011 (PMC12114212; doi:10.3390/microorganisms13051011)

Figure S1 The correlations between multiple dimensions diversity. FunFunc, FunPhy and FunRich are the functional, phylogenetic and taxonomic diversity of fungi, respectively. BacFunc, BacPhy and BacRich are the functional, phylogenetic and taxonomic diver-sity of bacteria, respectively. VegFunc, VegPhy and VegRich are the functional, phylogenetic and taxonomic diversity of plant, respectively.

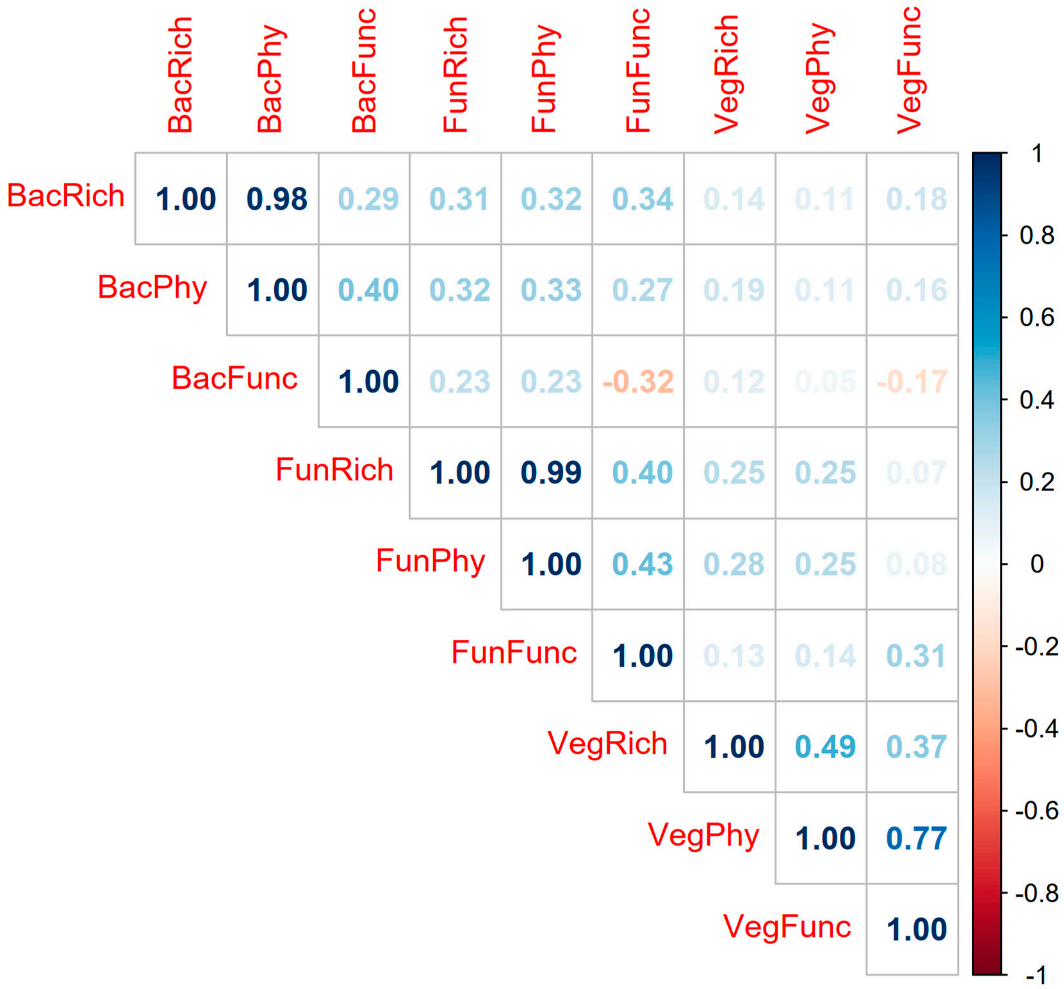

Supplement: Supplementary file 1 [file microorganisms-13-01011-s001.zip › microorganisms-3577826-supplementary.pdf]
